# Fractalkine/CX_3_CL1 protects striatal neurons from synergistic morphine and HIV-1 Tat-induced dendritic losses and death

**DOI:** 10.1186/1750-1326-6-78

**Published:** 2011-11-17

**Authors:** Masami Suzuki, Nazira El-Hage, Shiping Zou, Yun-Kyung Hahn, Mary E Sorrell, Jamie L Sturgill, Daniel H Conrad, Pamela E Knapp, Kurt F Hauser

**Affiliations:** 1 Department of Pharmacology and Toxicology, Virginia Commonwealth University School of Medicine, Richmond, VA 23298-0613 USA; 2 Department of Anatomy and Neurobiology, Virginia Commonwealth University School of Medicine, VA 23298-0709 USA; 3 Department of Microbiology and Immunology, Virginia Commonwealth University School of Medicine, VA 23298-0678 USA; 4 Institute for Drug and Alcohol Studies, Virginia Commonwealth University, Richmond, VA 2329-0310 USA; 5 Cancer Pathophysiology Division, National Cancer Center Research Institute (Tsukiji Campus), 5-1-1, Tsukiji, Chuo-ku, Tokyo 104-0045, JAPAN

**Keywords:** AIDS, opioid, heroin, drug abuse, glial cell, neuroAIDS, transgenic, cell death, microglia

## Abstract

**Background:**

Fractalkine/CX_3_CL1 and its cognate receptor CX_3_CR1 are abundantly expressed in the CNS. Fractalkine is an unusual C-X3-C motif chemokine that is important in neuron-microglial communication, a co-receptor for HIV infection, and can be neuroprotective. To assess the effects of fractalkine on opiate-HIV interactive neurotoxicity, wild-type murine striatal neurons were co-cultured with mixed glia from the striata of wild-type or *Cx3cr1 *knockout mice ± HIV-1 Tat and/or morphine. Time-lapse digital images were continuously recorded at 20 min intervals for up to 72 h using computer-aided microscopy to track the same cells repeatedly.

**Results:**

Co-exposure to Tat and morphine caused synergistic increases in neuron death, dendritic pruning, and microglial motility as previously reported. Exogenous fractalkine prevented synergistic Tat and morphine-induced dendritic losses and neuron death even though the inflammatory mediator TNF-α remained significantly elevated. Antibody blockade of CX_3_CR1 mimicked the toxic effects of morphine plus Tat, but did not add to their toxicity; while fractalkine failed to protect wild-type neurons co-cultured with *Cx_3_cr1*^-/-^-null glia against morphine and Tat toxicity. Exogenous fractalkine also normalized microglial motility, which is elevated by Tat and morphine co-exposure, presumably limiting microglial surveillance that may lead to toxic effects on neurons. Fractalkine immunofluorescence was expressed in neurons and to a lesser extent by other cell types, whereas CX_3_CR1 immunoreactivity or GFP fluorescence in cells cultured from the striatum of *Cx3cr1*^-/- ^(*Cx3cr1*^GFP/GFP^) mice were associated with microglia. Immunoblotting shows that fractalkine levels were unchanged following Tat and/or morphine exposure and there was no increase in released fractalkine as determined by ELISA. By contrast, CX_3_CR1 protein levels were markedly downregulated.

**Conclusions:**

The results suggest that deficits in fractalkine-CX_3_CR1 signaling contribute to the synergistic neurotoxic effects of opioids and Tat. Importantly, exogenous fractalkine can selectively protect neurons from the injurious effects of chronic opioid-HIV-1 Tat co-exposure, and this suggests a potential therapeutic course for neuroAIDS. Although the cellular mechanisms underlying neuroprotection are not certain, findings that exogenous fractalkine reduces microglial motility and fails to protect neurons co-cultured with *Cx3cr1*^-/- ^mixed glia suggest that fractalkine may act by interfering with toxic microglial-neuron interactions.

## Background

Opioid drugs can increase HIV replication and modify HIV pathogenesis through direct interactions with opioid receptor-expressing cells in the immune system [1-5]. We found that opioids can potentiate the neurodegenerative effects of HIV-1 in the central nervous system (CNS) through direct actions at μ-opioid receptor expressing neural cells [6-10], which has support from findings in nonhuman primates [2] and clinical studies [11,12]. The "opioid-cytokine connection" has been proposed to highlight the interrelatedness of the opioid and chemokine systems in HIV disease progression [13,14]. Not only can opioids potentiate the production of chemokines that are known mediators of HIV encephalitis, such as CCL5/RANTES and CCL2/MCP-1 [15-17], but opioid and chemokine systems can undergo mutual cross-desensitization [18,19], and opioid and chemokine receptors may interact directly at the molecular level through the formation of heterodimers [20-22].

Fractalkine (CX_3_CL1) [23] and its receptor CX_3_CR1 [24] are widely distributed within the nervous system [25,26] in rodents and humans. Unlike other chemokines, fractalkine and its receptor have a unique structural motif (CX_3_C) and are the only ligand-receptor pair within the CX_3_C subgroup. Accordingly, fractalkine does not cross-react with other chemokine receptors and CX_3_CR1 is not activated promiscuously by other chemokines [23,27,28]. Functionally, fractalkine is highly pleiotropic [23,27], acting as both an adhesion molecule and chemoattractant for T cells, NK cells, and macrophages [24,29,30]. CX_3_CR1 can also serve as an HIV-1 co-receptor with CD4 [31-33] and is hypothesized to facilitate the spread of HIV-1 infection [34]. Within the nervous system, fractalkine serves a major role as a membrane-tethered neuronal chemokine, while the *Cx3cr1 *gene is highly expressed by microglia [26,35]. There are reports suggesting that the receptor is also expressed by neurons and other glial types [25,26,36]. Importantly, emerging evidence indicates that when anchored to the neuronal plasma membrane, fractalkine can modify the actions of microglia. Depending on context, fractalkine can impart life-or-death signals through CX_3_CR1. CX_3_CR1 engagement can restrict the aggressiveness of activated microglia [37,38], or can protect neurons from exposure to HIV-1 Tat [39] or gp120 through the activation of Akt/PKB [36]. Alternatively, *Cx3cr1 *gene deletion has been shown to limit microglial-mediated neuron death in Alzheimer's disease [40].

Fractalkine levels are increased in HIV-associated neurocognitive disorders (HAND) [41-43] and in pediatric patients with HIV-1 encephalitis [39]. Increased fractalkine expression has been proposed as a both a mechanism of compensatory neuroprotection as part of a generalized response to lentiviral infections in the CNS [39,44] and as contributing to the neuronal injury without affecting neuronal death in neuroAIDS [43]. By contrast, other investigators report reductions in fractalkine and CX_3_CR1 levels and signaling in neuroAIDS [45]. More recently, fractalkine has been proposed as important for trafficking HIV-infected lymphocytes into the brain [34,42]. Soluble fractalkine attenuates gp120 toxicity in hippocampal neuron cultures in the presence or absence of a glial feeder layer suggesting a direct neuroprotective role [46]. Fractalkine can also modify neuronal signaling through β1-integrins [47]. Interestingly, Tat possesses an Arg-Gly-Asp (RGD) domain [48] and can bind to α5β1 integrin [49], as well as several other integrins including αvβ3 and αvβ5 [49-51], which suggests at least one possible molecular site where fractalkine and HIV-1 Tat might interact.

Fractalkine injections into the periaqueductal grey attenuate the antinociceptive effects of μ, δ, or κ opioid receptor activation, while fractalkine itself has no effect, suggesting heterologous cross-desensitization between CX_3_CR1 and several opioid receptor types [52]. Moreover, intrathecal administration of CX_3_CR1 blocking antibodies potentiates morphine analgesia, which was interpreted to result from the blockade of fractalkine-induced increases in IL-1β and inflammatory pain [53]. Activation of cannabinoid receptors with WIN55,212-2 reduced fractalkine expression by human astrocytes through actions at p38 MAPK that were attenuated by the type 2 cannabinoid receptor antagonist SR144528 [54]. By contrast, fractalkine does not appear to mediate methamphetamine-induced neurotoxicity [55].

These findings prompted us to ascertain whether disruptions in fractalkine-CX_3_CR1 signaling might underlie the exaggerated CNS pathology seen with opioid abuse-HIV-1 comorbidity. The data presented herein suggest that morphine and HIV-1 Tat co-exposure may contribute to neuronal injury by downregulating CX_3_CR1, and thus interfering with fractalkine signaling. Importantly, soluble fractalkine provided exogenously can rescue neurons from the accelerated injury and death caused by combined morphine and Tat in the presence of wild-type glia.

## Results

**Figure 1:**
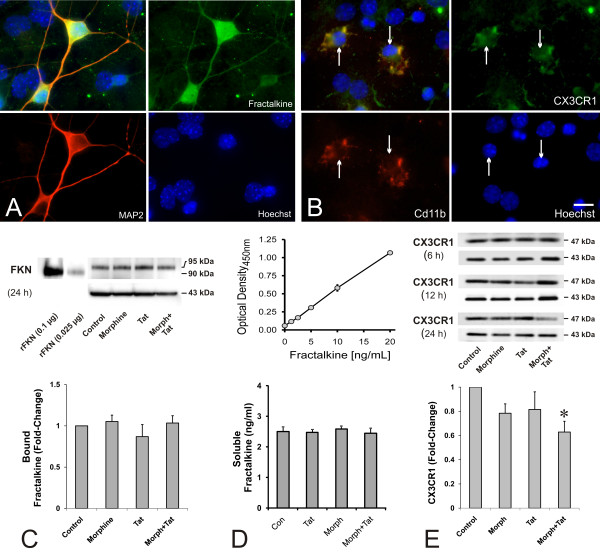
**Characterization and effects of morphine and/or HIV-1 Tat exposure on bound and soluble fractalkine, and CX_3_CR1 levels in mixed striatal neuron-glia cultures**. Fractalkine immunofluorescence was readily co-localized in MAP2-positive neuron cell bodies and dendrites (A), while a majority of the CX_3_CR1 immunoreactive cells were CD11b-positive microglia (B); scale bar = 15 μm. Fractalkine derived from neuron-glial co-cultures presented a prominent band at approximately 95-kDa (C). This is slightly higher than the non-glycosylated, recombinant fractalkine (rFKN) control protein detected at 90-kDa; 0.1 μg and 0.025 μg fractalkine (FKN) were loaded onto the left-hand lanes. The relative abundance of either variant appeared to be unaffected by morphine (Morph) and/or Tat treatment at 24 h (C); n = 3 experiments. The amount of fractalkine released into the culture medium was unaffected following 2 h of morphine and/or Tat exposure (D); the limit of fractalkine detection by ELISA was 320 pg/ml (standard curve in D); n = 3 experiments. Unlike fractalkine, treatment with morphine and Tat together significantly reduced CX_3_CR1 levels (~47-kDa) at 24 h, but not 6 h or 12 h, compared to vehicle-treated controls (E) (*P < 0.02 vs. vehicle-treated controls; Kruskal-Wallis ANOVA and nonparametric post hoc tests), while CX_3_CR1 levels were unaffected by exposure to morphine or Tat alone (E); fractalkine and CX_3_CR1 levels were normalized to actin (~43-kDa bands in C,E) in immunoblots; n = 6 experiments.

The distribution of fractalkine immunofluorescence suggested that the chemokine was primarily expressed by neurons, although other cell types occasionally displayed fractalkine immunoreactivity, including subsets of astrocytes as previously reported [25,56]. In untreated control cultures, robust fractalkine immunoreactivity is evident in MAP2-immunopositive neurons (Figure 1A). In contrast to fractalkine, the most intense CX_3_CR1 immunofluorescence was punctate and associated with CD11b-immunoreactive microglia (1B; arrows). Preabsorbed fractalkine controls displayed no specific immunoreactivity (not shown).

### Effect of Tat and morphine on levels of fractalkine and CX_3_CR1

To detect fractalkine by immunoblot, we used an antibody directed against the N-terminal chemokine domain (AF472; R&D Systems, Minneapolis, MN). In immunoblots of whole-cell lysates, which include neurons, astroglia and microglia, fractalkine presented as an approximately 95-kDa band (Figure 1C). Recombinant, non-glycosylated fractalkine, which served as a positive-control, resulted in a 90-kDa band [57]. The relative abundance of either variant appeared to be unaffected by morphine and/or Tat treatment at 24 h (Figure 1C). The antibody does not recognize the residual approximately 20 kDa C-terminal tail that anchors fractalkine to the cell membrane.

To assess whether morphine and/or Tat treatment affected fractalkine shedding, the amount of released fractalkine in the conditioned medium of mixed neuron-glial cultures was analyzed by ELISA at 2 h following morphine and/or Tat exposure (Figure 1D). The limit of detection of the fractalkine assay was 320 pg/ml. Soluble fractalkine was detected in the medium of vehicle-treated control cultures (2.50 ± 0.15 ng/ml), suggesting that fractalkine is constitutively cleaved and released at relatively high levels. The proportion of neurons in our cultures were purposely low relative to numbers of glia (1:20 ratio) to mimic neuron:glia ratios in vivo. Both astroglia and neurons express fractalkine [25,36,41,54,56,58]. Despite reports that a vast majority of fractalkine originates from neurons (especially when they are co-cultured with glia [59]), the neurons in our cultures are vastly outnumbered by astroglia. Thus, while relatively constant amounts of fractalkine are released irrespective of morphine and/or Tat exposure, it is difficult to know the neuronal versus astroglial contribution or the extent to which more subtle, subcellular changes in neuronal release might be masked. Thus, the results are inconclusive regarding whether changes in fractalkine shedding by neurons underlie synergistic morphine-Tat neurotoxicity (Figure 1C,D), and additional study is warranted.

To explore possible morphine and/or Tat-induced alterations in fractalkine receptor expression by microglia, CX_3_CR1 was examined by immunoblotting. In mixed striatal cultures, CX_3_CR1 was detected as a major band at about 47 kDa, which coincides with its expression pattern in other cell types [29]. Compared to vehicle-treated controls, CX_3_CR1 levels were diminished, although not significantly, in either morphine or Tat-exposed cultures at 24 h (Figure 1E). Treatment with morphine and Tat together did significantly reduce CX_3_CR1 levels by about 40% compared to controls, but not compared to cultures treated with morphine or Tat alone. The findings show that, in combination, morphine and Tat may cumulatively downregulate CX_3_CR1 levels (Figure 1E). CX_3_CR1 expression was largely restricted to microglia in our striatal cultures. Accordingly, the marked decline in CX_3_CR1 levels with morphine and Tat co-exposure is presumed to represent a preferential microglial response. It is also noteworthy that reductions in CX_3_CR1 levels occurred despite previous findings that Tat (14.7 ± 1.3%) or morphine plus Tat (13.7 ± 1.7%) treatment significantly increases the proportion of microglia in mixed-glial cultures from striatum compared to controls (8.8 ± 0.6%) [60].

### Neurotoxicity

**Figure 2:**
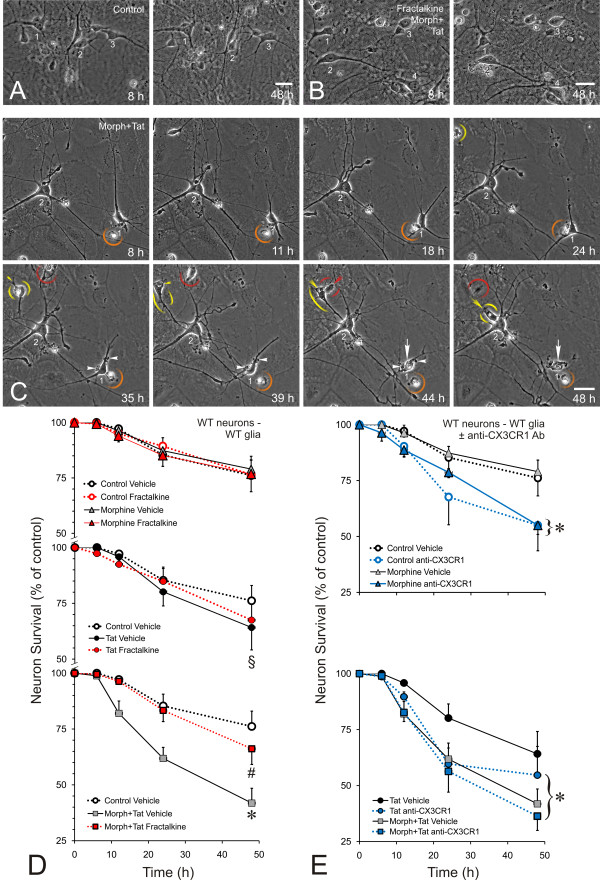
**Effects of fractalkine on morphine and/or HIV-1 Tat-induced toxicity was repeatedly tracked in individual striatal neurons (A-E)**. A within-subjects design and computer-assisted, time-lapse tracking of individual neurons is used to compare the survival of the same neuron before and at 20 min intervals throughout treatments (A-E). Thriving neurons are evident in control (A; neurons 1-3) or morphine (Morph) + Tat-treated cultures co-administered fractalkine (B; neurons 1-4) at 48 h. By contrast, increased neurodegeneration and death was apparent with morphine plus Tat exposure (C). Neuron death is often preceded by the systematic degeneration of neurites and fragmentation of the cell body (Tat + morphine treatment; arrowheads, neuron 1), while death per se occurs precipitously; denoted here by fragmentation of the cell body and some loss of birefringence (arrow, neuron 1; 44 h and 48 h) (C). Some individual microglia that were tracked are encircled in red, orange, and yellow, respectively (arrowheads indicate prior movement); there was close association of microglia with the dying (1) and surviving (2) neuron (not all microglia in the fields are encircled) (C). Neuron death has been confirmed using other viability markers (see text). Many neurons remain viable despite morphine and Tat co-exposure (e.g., neuron 2); some surrounding cells, including astroglia, immature glial precursors and microglia, can display sporadic movement; scale bars = 20 μm. While Tat alone did not cause significant neurotoxicity relative to controls by 48 h [61,62]; there was a significant interaction when overall neuron losses were compared in Tat versus non-Tat vehicle-treated cultures (^§^*P *< 0.03; Tat × time vs. Vehicle-control × time). Compared to controls or exposure to fractalkine alone, combined morphine and Tat (Morph+Tat) treatment increased neuronal death at 48 h (D) (*P < 0.05 vs. controls). Exogenous fractalkine (1 μg/ml) (red markers and connecting lines) prevented the accelerated neuronal death caused by combined Morph+Tat (^#^*P *< 0.05 vs. Morph+Tat) (D). By contrast, CX_3_CR1 immunoblockade (blue markers and connecting lines) by itself caused significant neurotoxicity that was indistinguishable from the combined effects of Morph+Tat (E) (*P < 0.05 vs. controls). Moreover, when CX_3_CR1 blockade was combined with Morph+Tat, there were no additive neuron losses (E). The findings suggest a critical role for fractalkine-CX_3_CR1 signalling in potentiating the toxic effects of opioids in Tat-exposed neurons; data are the mean number of surviving neurons (compared to pretreatment numbers) ± SEM from n = 3-6 experiments; wild-type (WT) neurons and mixed glia; antibodies (Ab); scale bar = 20 μm.

Analyzing the response of individual neurons throughout the experiment eliminates inter-subject variability and permits subtle treatment effects to be revealed [61,62]. Computer-aided tracking permits large numbers of neurons (and glia) to be followed and consistently assessed over time, which greatly facilitates and improves the sensitivity of the assay (Figure 2A-E). Although Tat alone did not cause significant neurotoxicity relative to controls at individual times tested, there was a significant interaction effect (^§^*P *< 0.03; Tat × time vs. Control × time) indicating a significantly greater decline in the rate of survival of Tat-treated neurons compared to controls from 0 h to 48 h (Figure 2D). This suggests that even greater proportions of neurons are likely to be lost with more prolonged Tat exposure, as observed previously [62]. Alternatively, combined exposure to Tat and morphine caused interactive neuron losses (Figure 2C-D), similar to the accelerated synergistic neuron death described previously with morphine and Tat co-administration [10] (*P < 0.05) (Figure 2D). These interactive effects have previously been shown to be selective for Tat, and do not occur with either denatured or mutant forms of the viral protein [10,61].

### Fractalkine rescues neurons from synergistic morphine and Tat neurotoxicity

Compared to vehicle controls, neuron viability was unaffected by treatment with fractalkine (1 μg/ml) alone (Figure 2D). By contrast, co-exposure to morphine (500 nM) and Tat (100 nM) significantly enhanced neuron losses during the 48 h exposure period (Figure 2C,D), while the synergistic neurotoxicity was entirely prevented by addition of exogenous fractalkine (1 μg/ml) to the medium (Figure 2B,D). Incubation with CX_3_CR1 blocking antibodies alone was also neurotoxic, at levels indistinguishable from the combined effects of morphine and Tat (Figure 2E). However, when anti-CX_3_CR1 antibodies were co-incubated with morphine and Tat, there was no additional neurotoxicity (Figure 2E). This suggests that neuron death is enhanced when protective signaling through CX_3_CR1 is attenuated, either by reducing levels of CX_3_CR1 (as seen after 24 h with morphine and Tat co-exposure; Figure 1E), or by physically limiting CX_3_CL1-CX_3_CR1 interactions through immunoblockade (Figure 2E). Combining CX_3_CR1 blockade with morphine and Tat exposure does not increase the amount of neuron death seen with morphine and Tat co-administration alone, perhaps indicating that CX_3_CR1 function is already maximally reduced in morphine plus Tat exposed cells. While Tat or morphine by themselves did not significantly reduce CX_3_CR1 levels, the trend towards reduced expression (Figure 1E) suggests that this might occur with more prolonged exposure.

### Selectivity of fractalkine's neuroprotective action

**Figure 3:**
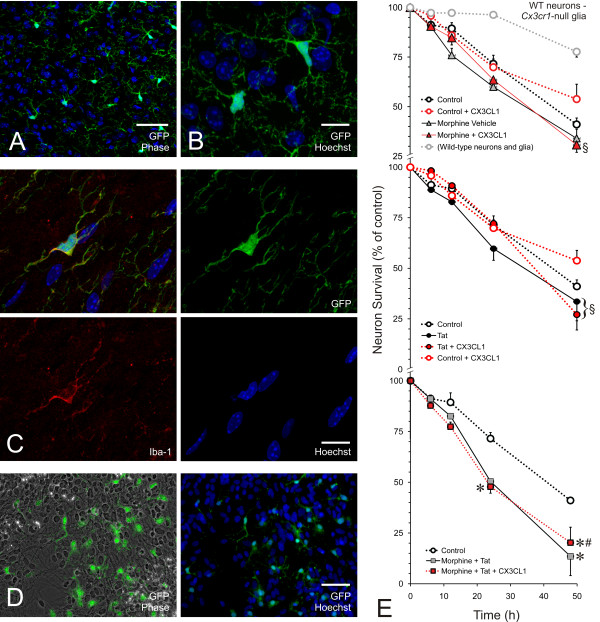
**Studies with *Cx3cr1*^-/- ^mice: Fluorescent images and effects of *Cx3cr1*^-/- ^glia on fractalkine-mediated neuroprotection**. Images of the striatum (A-C) and of mixed glial cultures (D) from *Cx3cr1*^-/- ^mouse striata. Microglia within the striatum of *Cx3cr1*^-/- ^(*Cx3cr1*^GFP/GFP^) mice fluoresce green (A,B) and are Iba-1 immunoreactive (C); Hoechst counterstained nuclei (blue); scale bars = 50 μm (A) and 10 μm (B,C). Robust GFP expression is maintained when the cells are placed into culture (D). As can be appreciated from the Hoechst stain, the CX_3_CR1-expressing population is significant although the majority of the cells in the culture lack CX_3_CR1. The loss of CX_3_CR1 on glia has a dramatic effect on the ability of fractalkine to protect morphine and Tat-treated striatal neurons (E). Neurons from the striata of wild-type mice were plated onto mixed glia prepared from *Cx3cr1*^-/- ^mouse striata and followed over 48 h using our standard paradigm. In general, wild-type neurons co-cultured with *Cx3cr1*^-/- ^glia appeared to be less viable than with wild-type glia (upper panel; compare gray and black dotted lines); however, these groups were not compared statistically since the experiments were not run concurrently. The neuroprotective effects of fractalkine (CX_3_CL1; 1 μg/ml) are abolished when wild-type neurons are co-cultured with *Cx3cr1*^-/- ^mixed glia. Under these conditions, fractalkine no longer protects neurons from combined morphine and Tat exposure (E, lower panel; *P < 0.05 vs. vehicle- or fractalkine-treated controls). There were no differences in survival between controls ± fractalkine. Addition of fractalkine did not change the toxicity of morphine, Tat, or morphine + Tat treatments. There is, however, some possibility that fractalkine enhances baseline neuron survival in the *Cx3cr1*^-/- ^glial co-cultures. This was indicated by significant differences only at 48 h between Tat + fractalkine versus control + fractalkine survival (^§^*P *< 0.05), even though survival did not differ when comparing vehicle + control vs. fractalkine + control or vehicle + Tat vs. fractalkine + Tat treatments. A similar inconsistency was observed only at 48 h with morphine treatments (^§^*P *< 0.05).

To explore the selectivity of fractalkine's neuroprotective effect and to begin to discern whether fractalkine is acting via neuronal or glial receptors, *Cx3cr1*^-/- ^(*Cx3cr1*^GFP/GFP^) mice were obtained and mixed glial cultures established from their striata. Prior to starting in vitro studies, *Cx3cr1*^-/- ^mice were examined histologically. In the striatum, the predominant GFP^+ ^cell type was microglia, which were intensely fluorescent (Figure 3A,B). The intensely fluorescent GFP^+ ^cells readily co-localized with ionized calcium binding adaptor molecule 1 (Iba-1) (Figure 3C), as previously reported for this *Cx3cr1*^-/- ^mouse strain [38], and high levels of GFP fluorescence were retained by microglia, but not astrocytes, in mixed glial cultures (Figure 3D) (Table 1).

**Table 1:** Effects of morphine and/or HIV-1 Tat exposure ± fractalkine on the percentage of microglia in mixed-glia cultures from striata of *Cx3cr1*^-/- ^(*Cx3cr1*^GFP/GFP^) mice (^‡^).

	Control	Morphine	Tat	Morphine + Tat
**Microglia (%) ** *(Cx3cr1^-/- ^mixed glia) *	31.0 ± 6.7^§^	26.7 ± 2. 5	26.7 ± 5.1	34.9 ± 1.6

**Microglia (%)** *(Cx3cr1^-/- ^mixed glia + fractalkine)*	25.8 ± 2.3	25.8 ± 6.7	25.5 ± 4.0	30.9 ± 0.7

^‡ ^No significant effects of morphine and/or HIV-1 Tat ± fractalkine (1 μg/ml) exposure on the proportion of GFP^+ ^microglia were noted; effects were compared using 2-way ANOVA.

^§ ^The proportion of microglia in mixed glial cultures from Cx3cr1^-/- ^mice was much greater than percentages seen in equivalent striatal cultures from wild-type mice (e.g., 8.8 ± 0.6% Iba-1^+ ^microglia in control cultures) determined as part of another study [60].

In cell cultures, *Cx3cr1*-null microglia differed considerably from their wild type counterparts. The proportion of microglia:astrocytes was over 3-fold greater than that consistently seen in wild-type mixed-glial cultures [60], despite identical culture conditions in the present study. When wild-type neurons were co-cultured with *Cx3cr1*^-/- ^glia, baseline levels of neuron death (41.0 ± 3.2% surviving) appeared to be much greater than losses typically seen in the presence of wild-type glia (Figure 3E, upper panel; 77.7 ± 2.7% surviving). Fundamental differences in cellular makeup and behavior of mixed-glia cultures from *Cx3cr1*-null compared to wild type cultures, even prior to the addition of neurons, negated the validity of direct "side-by-side" comparisons of neuron survival in co-culture with wild-type versus *Cx3cr1*-null mixed glia. However, in our extensive experience with this co-culture model [60,63,64], neuron survival rates in control cultures are always between 75-90% at 48 h. The greater apparent neuron losses might be related to the > 300% increase in microglial numbers observed in*Cx3cr1*^-/- ^cultures (Table 1). Importantly, the relative neuroprotective effects of fractalkine (1 μg/ml) against morphine + Tat neurotoxicity were abolished when wild-type neurons were co-cultured with *Cx3cr1*^-/- ^mixed glia (Figure 3E), suggesting fractalkine protection was mediated by CX_3_CR1-expressing microglia. Although there were no differences in survival between vehicle + control versus fractalkine + control neurons, there is some possibility that fractalkine enhances baseline neuron survival in the *Cx3cr1*^-/- ^glial co-cultures. This is suggested by significant differences only at 48 h between Tat + fractalkine versus control + fractalkine survival (^§^*P *< 0.05), even though survival does not differ within control ± fractalkine or Tat ± fractalkine treatments. A similar inconsistency is observed only at 48 h with morphine treatments (^§^*P *< 0.05). The inconsistencies may result from some residual actions in the wild-type cells since even the most highly enriched neuron cultures contain some (< 1%) glia.

### Effect of fractalkine on microglial motility

**Figure 4:**
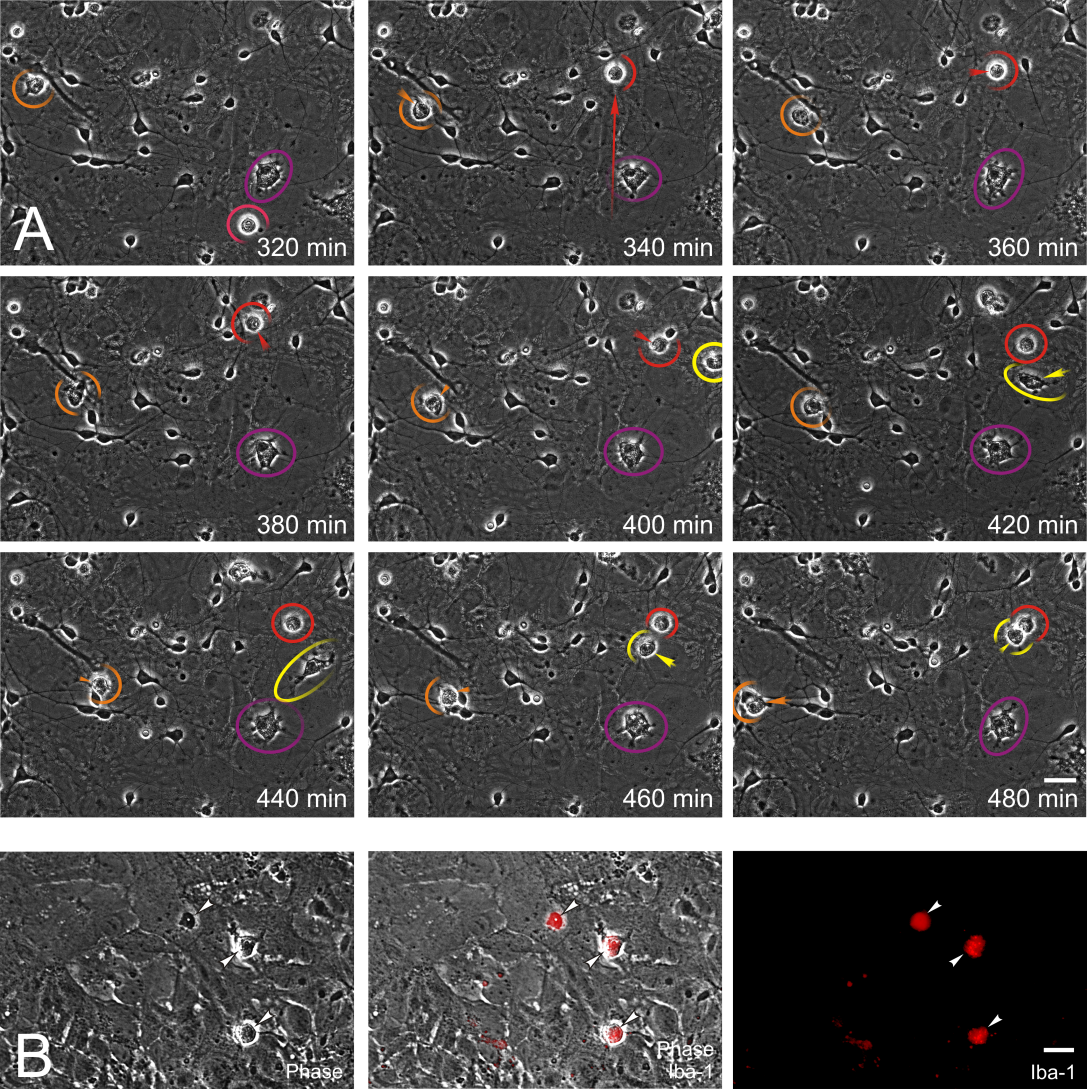
**Computer-assisted, time-lapse tracking of individual microglial cells (A-B)**. Individual microglia were tracked using phase-contrast optics (encircled/partially encircled and color-coded), their vectorial movement indicated by the direction and length of arrows/arrowheads (A), and their identity consistently confirmed by the specific microglial marker Iba-1 (B); scale bar = 20 μm.

**Figure 5:**
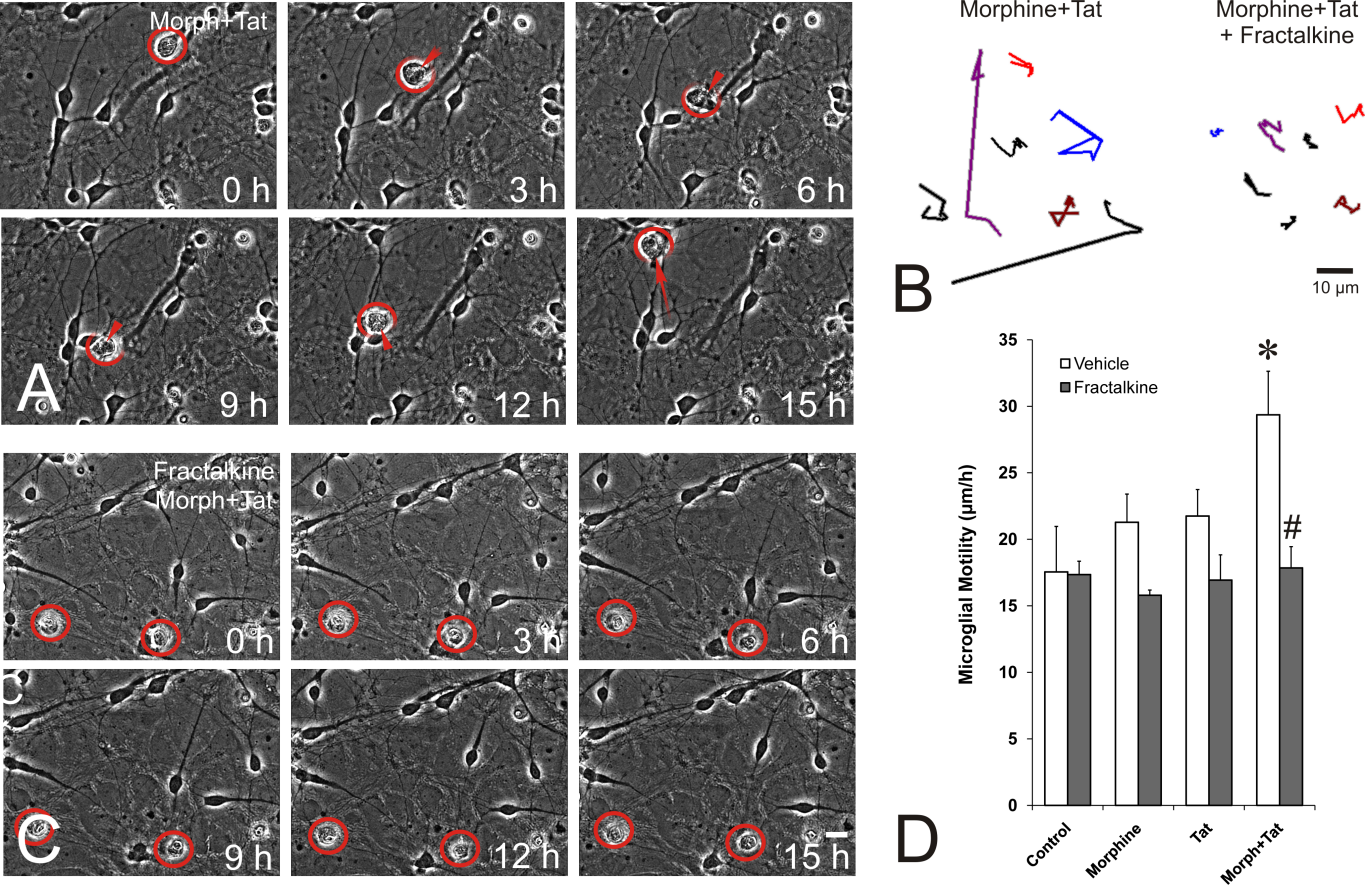
**Effects of fractalkine on morphine and/or Tat-induced alterations in microglial motility (A-D) and nitrosative stress (E-F)**. Morphine and Tat together (Morph+Tat) increased microglial movement in time-lapse images (representative cells circled in red) (A,B), while concurrent fractalkine exposure negated the effects of Morph+Tat (representative cells encircled in red) (B,C). When the vectorial trajectories of individual microglial movement are plotted each hour for 6 h, (i) the sporadic movements of individual cells and (ii) the ability of fractalkine to attenuate the overall motility are appreciated (B). Measurements confirmed that combined Morph+Tat increased microglia motility compared to vehicle, morphine, or Tat exposure alone (*P < 0.05). Addition of exogenous fractalkine (1 μg/ml) completely reversed the effect of Morph+Tat (D) (^#^*P *< 0.05 vs. Morph+Tat alone), returning motility to baseline levels. The average movement of ~100 microglia in each treatment group was determined by measuring the distance from nuclear position at 0 h vs. 12 h per treatment in each experiment; values are the mean movement (μm/h) ± SEM from n = 4 experiments. Representative microglia that were tracked are encircled in red; the occasional macrophage/microglial interlopers that appeared and/or disappeared within the observation field and could not be tracked are circled in yellow; scale bar = 20 μm.

Amoeboid migration as seen in leukocytes is an active process requiring actin polymerization, which is typically signaled through small GTPases in response to extracellular stimuli [65]. The chemotactic targeting of infected or injured cells is a critical aspect of microglial function as immune effectors. Accordingly, we investigated the effects of fractalkine on microglial movement. The identity of individual activated microglia was readily discerned using phase-contrast microscopy by criteria described in the Methods, and the identity of individual cells could be interrogated using immunofluorescent-tagged antibodies against surface antigens such as Iba-1 shown in Figure 4A-B. Movement differed among individual microglia (Figures 4A &5A-C), though the average movement within a population was quite reproducible and highly sensitive to extracellular signals (Figure 5A-D). We measured the linear movement of individual microglia each hour from 0 to 12 h after treatment. Vehicle-treated microglia migrated an average of 17 μm/h during this time and motility in microglia that were not exposed to Tat and/or morphine was unaffected by exogenous fractalkine (Figure 5D). Treatment with morphine or Tat alone tended to increase microglial movement, but the effect was not significant over 12 h. By contrast, continuous exposure to combined morphine and Tat significantly increased the movement of microglia to ~30 μm/h. In this case, fractalkine (1 μg/ml) significantly inhibited the increase in microglial migration rate, bringing it back to pretreatment levels (~17 μm/h) (Figure 5D). The fact that fractalkine did not reduce baseline motility, and did not reduce motility increases due to Tat+ morphine to below pretreatment values, suggests that motility is only partially controlled by fractalkine and that alternative signals [66,67] maintain baseline rates of movement.

### Effects of morphine and/or Tat ± fractalkine on dendrite length

**Figure 6:**
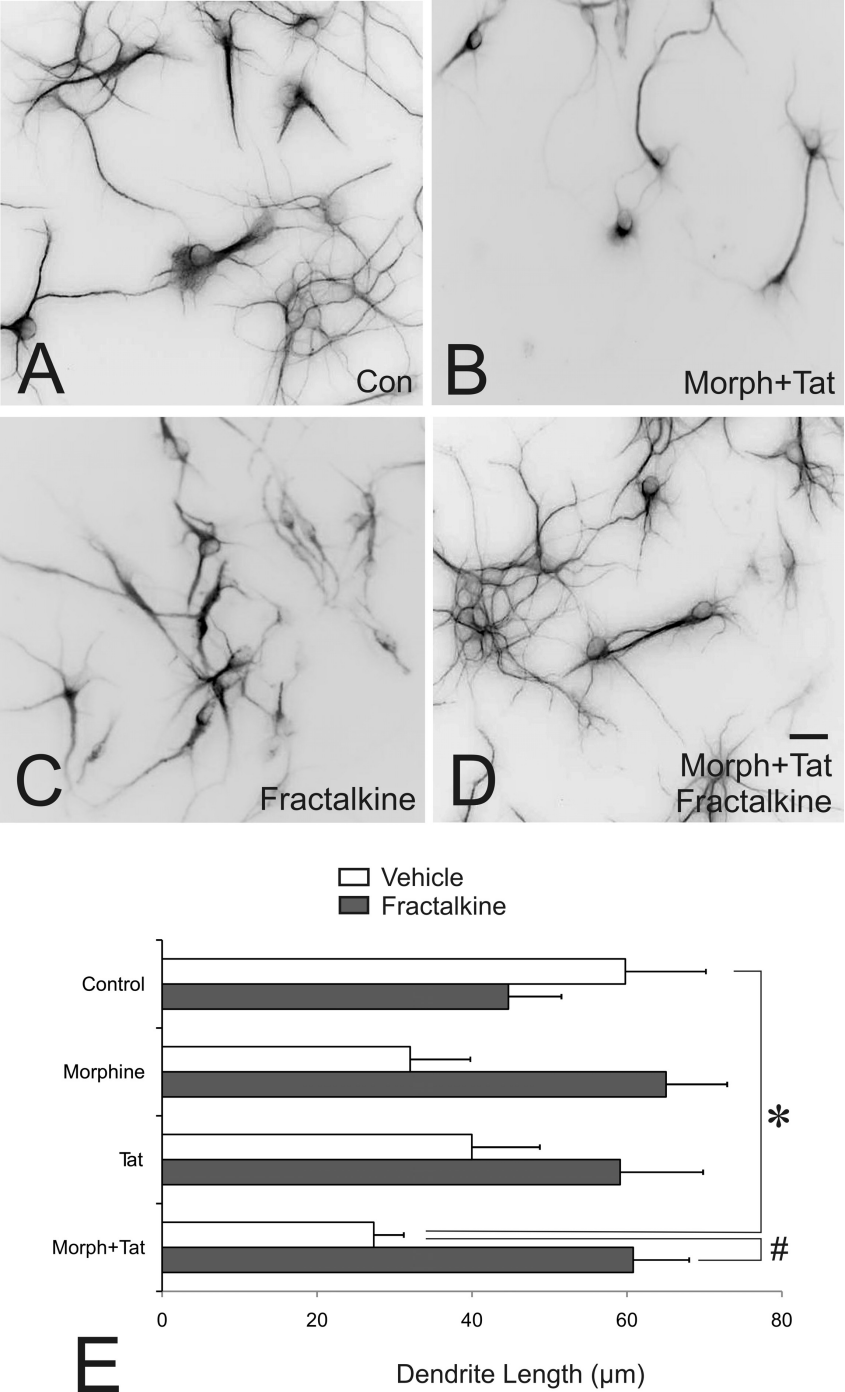
**Effects of fractalkine or immunoneutralizing anti-CX_3_CR1 antibodies on morphine and/or Tat-induced degeneration of MAP2-immunoreactive dendrites of striatal neurons at 72 h (A-E)**. Compared to vehicle controls (Con) (A), morphine and Tat (Morph+Tat) (B) caused significant reductions in dendritic length (*P < 0.05 vs. vehicle controls) (E). Fractalkine prevented Morph+Tat-induced dendritic pruning (^#^*P *< 0.05 vs. Morph+Tat) (D,E), but by itself had no effect (C,E). Data in E are mean dendrite length (μm) ± SEM from n = 4 experiments; scale bar = 20 μm.

There was a tendency for morphine or Tat alone to reduce dendrite length following 72 h exposure (Figure 6A-E), but the effects were not significant (Figure 6E). By contrast, morphine and Tat in combination caused significant reductions in dendritic complexity in MAP2-immunoreactive striatal neurons after 72 h (Figure 6B,E). Importantly, concurrent exposure to exogenous fractalkine (1 μg/ml) prevented the dendritic pruning caused by morphine and Tat co-exposure (Figure 6D,E).

### Effects of fractalkine on the production of TNF-α

**Figure 7:**
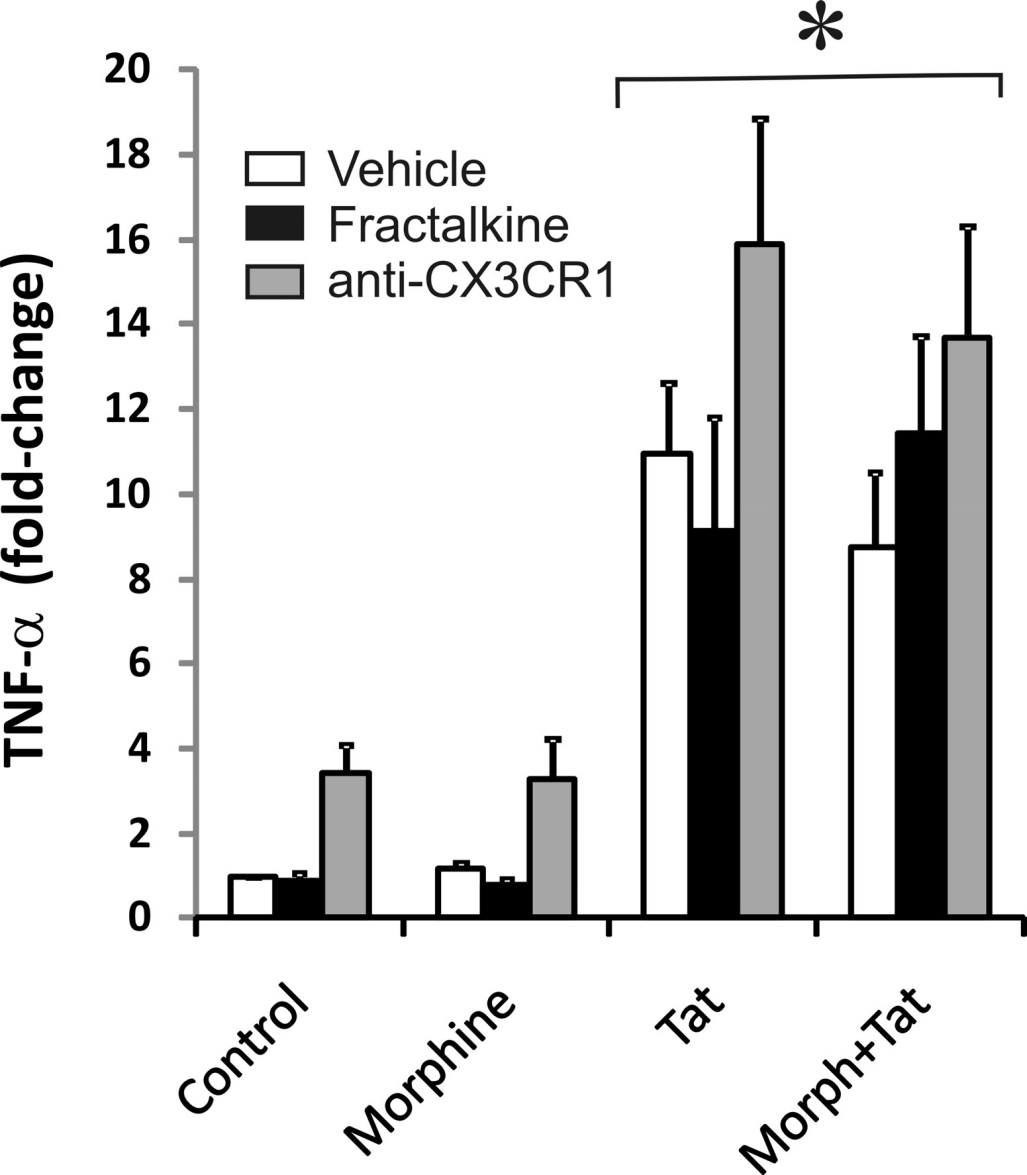
**Effects of fractalkine or immunoneutralizing anti-CX_3_CR1 antibodies on morphine and/or Tat-induced TNF-α production by mixed neuron-glial cultures at 12 h**. Tat ± morphine (Morph) significantly increased TNF-α production by mixed neuronal-glial cultures from striatum (*P < 0.05 vs. vehicle-treated controls); Tat ± morphine-induced TNF-α production was unaffected by fractalkine or CX_3_CR1 immunoblockade, although there was a trend toward increased TNF-α release in control and morphine-treated cultures with CX_3_CR1 blockade.

TNF-α has been shown to play a central role in initiating inflammatory cascades in Tat exposed astroglia and microglia [15,68,69]. ELISA was performed at 12 h following morphine and/or Tat exposure to determine whether fractalkine might be protecting neurons by reducing TNF-α levels in our cultures. As shown before for striatal astroglia [17,70], Tat ± morphine significantly increased TNF-α production by these mixed neuronal-glial cultures (Figure 7); TNF-α production was unaffected by fractalkine or immunoblockade of CX_3_CR1, although there was a trend toward increased TNF-α release in control and morphine-treated cultures with CX_3_CR1 blockade (Figure 7). Thus, fractalkine-mediated protection occurs despite a sustained TNF-α-induced inflammatory signal.

## Discussion

Our results indicate that exogenous fractalkine completely blocks the synergistic neurotoxicity caused by combined morphine and Tat exposure. This appears to be mediated by CX_3_CR1 expressed by microglia, since fractalkine neuroprotection is abolished when wild-type neurons are co-cultured with *Cx3cr1*-null mixed glia and because CX_3_CR1 is almost exclusively expressed by microglia in these cultures. Moreover, the neuroprotection is accompanied by a marked reduction in microglial motility that was otherwise significantly increased by morphine and Tat co-exposure. Although exogenous fractalkine is able to (i) rescue opiate and HIV-1 Tat treated neurons and (ii) limit microglial motility, the present study does not fully discern the cellular sites or mechanisms of fractalkine's protective action. Thus, while the neuroprotective effects of fractalkine are presumed to be due to direct actions on striatal microglia in the present study, we cannot completely rule out the possibility that fractalkine can act via alternative cell types in other brain regions [36,71].

Because the neurotoxic effects of HIV Tat and opiates were negated by exogenous fractalkine, we questioned whether fractalkine-CX_3_CR1 signaling might be disrupted in neurons and/or glia exposed to opiates and Tat and began to address this question in a preliminary fashion. A disruption in fractalkine-CX_3_CR1 signaling by opiate-HIV protein exposure would parallel evidence from a variety of other CNS disorders where fractalkine-CX_3_CR1 interfacing appears to be altered, including Parkinson's and Alzheimer's diseases, as well as in HIV dementia [40,44,72]. Close associations between fractalkine-expressing neurons and CX_3_CR1-positive microglia have been described in the CNS of pediatric patients with HIV-1 encephalitis [39], but expression levels of either the fractalkine ligand or receptor were not ascertained. More recently, CX_3_CR1-directed pruning of aberrant synapses was found to be necessary for normal CNS maturation [73]. *Cx3cr1*-null mice retained large numbers of immature synapses displaying deficits in neuronal function and behavior [73]. Assuming restricted fractalkine-CX_3_CR1 interfacing, an attenuated signal might originate from a decline in fractalkine, its receptor or both.

Fractalkine has multiple neuroprotective, cell-adhesion, and chemotactic functions through actions as both a bound and a diffusible factor [37,38]. This complexity adds to the challenge of understanding fractalkine-CX_3_CR1 actions. Membrane-bound fractalkine can function as an adhesion molecule for cells that express CX_3_CR1, while proteolytically released fractalkine functions as a more typical soluble chemokine [30]. Fractalkine is reportedly cleaved by ADAM (a disintegrin and metalloproteinase) 10 and perhaps ADAM17 (TNF-α converting enzyme or TACE) [57,74-76]. Cleavage results in soluble 60-85-kDa fractalkine (depending on residual glycosylation) and a ~20-kDa transmembrane C-terminal fragment that remains anchored to the cell membrane [57]. Once shed, fractalkine undergoes tissue-specific processing, which may contribute to localized immune responses [77,78]. Soluble fractalkine limits microglial neurotoxicity [30,36,38], as seen in the present study, and sustained fractalkine release may be necessary for continued protection from microglial surveillance [59].

In control cultures, the presence of secreted fractalkine in the medium suggests that bound fractalkine is constitutively cleaved and tonically released by ADAM10 [74]. ADAM10 is inducible and is acutely sensitive to increases in [Ca^+2^]_i _caused by excitotoxic levels of glutamate [76]. Despite the potential for dynamic changes in fractalkine release, the concept that morphine ± HIV-1 Tat exposure neurotoxicity might be mediated by altered fractalkine production or shedding is not supported by our findings. The relative abundance of bound (immunoblots) and released, soluble fractalkine (ELISA) was unaffected by morphine or Tat treatments. Nevertheless, additional studies are needed to address this question systematically. Although neurons reportedly shed greater amounts of fractalkine than astroglia [59], because astroglia outnumber neurons in our cultures by ~20-fold, the relative contribution of each to the fractalkine pool is uncertain and a potentially unique response by neurons may be overshadowed.

There is less agreement regarding how CX_3_CR1 levels affect neuron survival or function. CX_3_CR1 is most often reported as a ~50-kDa protein [79], which is in agreement with the present findings, although cell-type specific CX_3_CR1 isoforms of ~40 kDa have been reported [80] that likely result from alternative splicing and/or differences in CX_3_CR1 glycosylation [79]. The cellular distribution and intensity of CX_3_CR1 immunoreactivity and GFP in *Cx3cr1*-null mice indicated that the receptor was expressed by microglia at high levels in striatum (Figures 1B &3A-D). In cultures of mixed glia lacking the *Cx3cr1 *gene, we found the proportion of microglia:astroglia was much higher. Moreover, when wild-type neurons were co-cultured with *Cx3cr1*-null glia, the background rate of neuron death was much greater than with wild-type glia--even under identical culture conditions. This suggests fundamental differences between the function of *Cx3cr1*-deleted and wild-type microglia. The neuroprotective effects of fractalkine were lost when morphine and Tat-co-exposed wild-type neurons were cultured with *Cx3r1*-knockout glia. This strongly infers that fractalkine protects neurons through interactions with microglial CX_3_CR1, although inherent differences in the cytotoxicity of *Cx3cr1*-deleted microglia may represent a confound. Unlike fractalkine, CX_3_CR1 levels showed a classic downregulation after 24 h continuous exposure to combined morphine and Tat. The reduction appeared to be additive since morphine and Tat by themselves both produced modest, but not significant, reductions in CX_3_CR1 levels. Antibody blockade of CX_3_CR1 caused toxicity equal to Tat and morphine co-exposure, while *Cx3cr1 *gene deletion from glia appeared to increase baseline levels of neuron death even in control cultures (see Figure 3E).

Microglial chemotaxis, activation, and overactivation are regulated by hierarchical signals that are critically important in the inactivation/removal of pathogens [81]. Unchecked, microglial overactivation may cause bystander cytotoxicity [81-83]; yet, microglial activation appears reversible [81], suggesting an opportunity to allay the deleterious consequences of overactivation. HIV-1 or opioid exposure can trigger key events underlying microglial (and astroglial) activation including increases in oxyradicals [84], glutamate secretion [85], and the production of proinflammatory cytokines and chemokines [15-17,86]; while many of these deleterious signals can be attenuated by fractalkine/CX_3_CR1 activation [30,38,44,87,88]. In fact, increases in fractalkine may be a protective response in CNS injury and some diseases [89,90]. Our results suggest that CX_3_CR1 signaling can short-circuit key aspects of microglial overactivation caused by morphine and Tat, thereby enhancing neuron survival and function.

The above findings suggest that CX_3_CR1 signaling is fundamentally important for microglial function. In fact, it has been suggested that deleting the *Cx3cr1 *gene dysregulates so many aspects of microglial function (including functions regulating both protective and toxic bystander effects) that it may no longer be possible to correctly interpret the role of fractalkine-CX_3_CR1 interactions in a particular situation [30,37,38]. For example, neuron death was mitigated by *Cx3cr1 *deletion in models of Alzheimer's disease and ischemia [40,91]. By contrast, neurotoxicity was exacerbated by *Cx3cr1 *deletion in three other neuroinflammatory models [38], while CX_3_CR1 is essential for normal synaptic pruning during maturation [73]. Thus, the consequences of fractalkine-CX_3_CR1 signaling appear to be complex, varying depending on the particular disease, the brain region studied, and context. Clearly, the present findings suggest that CX_3_CR1 regulates critical microglial functions, and reductions in CX_3_CR1 levels coincide with basic imbalances in astroglial:microglial numerical matching and marked reductions in striatal neuron viability.

Notwithstanding its chemotactic and surveillance functions [28,37], fractalkine is reported to limit cytotoxicity by reducing microglial activation and killing [30,37,38]. HIV-1 Tat induces the production of chemokines that increase microglial chemotaxis including CCL2/MCP-1 [86,92] and CCL5/RANTES [15,16]. In the present study, although exogenous fractalkine completely blocked the increase in microglial motility caused by combined morphine and Tat exposure, it had no affect on baseline movement. Moreover, fractalkine negated the synergistic morphine and Tat neurotoxicity despite elevated levels of TNF-α in the culture medium. Assuming fractalkine may attenuate microglial activation through an alternative mechanism, we examined 3-nitrotyrosine and nitrite production, which are reactive nitrogen byproducts that are highly sensitive to morphine and HIV-1 Tat interactions [60,93]. In preliminary studies, we found that morphine + Tat-induced increases in both nitrosative byproducts were entirely unaffected by exogenous fractalkine (1 μg/ml) at 24 h or 48 h (data not shown). Additional studies are needed to assess whether fractalkine might affect other key redox and proinflammatory pathways in microglia before suggesting that fractalkine's actions are restricted to microglial motility or surveillance. Since nitrosative stress and TNF-α production lie upstream of, and instigate, multiple inflammatory signaling events, we propose that fractalkine selectively overrides key aspects of microglia activation at relatively late stages in the inflammatory process to prevent cytotoxic killing. If this assumption is correct the findings have considerable therapeutic implications for chronic substance abuse and HIV-1 comorbidity.

## Conclusions

Fractalkine-CX_3_CR1 signaling in striatal neuron-glia co-cultures appears to be compromised by opioid drug and HIV-1 co-exposure, and this may contribute to increased neuronal injury. Levels of CX_3_CR1 are reduced by morphine and Tat co-exposure, and the addition of exogenous fractalkine completely prevents the interactive neurotoxicity. Since CX_3_CR1 is expressed almost exclusively by microglia in the mixed-glial cultures, this suggests that fractalkine protects neurons against morphine and Tat co-exposure through direct actions on microglia. In glia from mice lacking the *Cx3cr1 *gene the proportion of microglia:astroglia was much higher and background rates of neuron death were greater than wild-type glia even under identical culture conditions, suggesting fundamental differences between the function of *Cx3cr1*-null and wild-type microglia. Despite these inherent differences, the neuroprotective effects of fractalkine were lost when morphine and Tat-co-exposed wild-type neurons are cultured with glia lacking the *Cx3r1 *gene further inferring fractalkine-CX_3_CR1 interactions as protective. Thus, fractalkine may have considerable therapeutic potential in limiting inflammation, microglial overactivation, and neuronal injury with opioid abuse and/or neuroAIDS.

## Methods

### Animals

ICR (CD-1) mice were purchased from Charles River Inc. (Charles River, MA), while *Cx3cr1*^-/-^(*Cx3cr1*^GFP/GFP^) mice [26] on a C57BL/6J genetic background were obtained from Jackson Laboratory (Bar Harbor, ME) and originally engineered by Dr. Dan Littman (Skirball Institute, New York University School of Medicine, NY, NY). In all experiments, mice were anesthetized and/or euthanized in accordance with NIH and local Virginia Commonwealth University IACUC guidelines, which minimize the number of mice used and their possible discomfort.

### Mixed-glial cultures

Mixed glial (astroglia and microglia) cultures were prepared from neonatal (P0-P1) wild-type ICR or *Cx3cr1*^-/- ^mouse striata (Charles River) as previously described [70]. Striata were dissected, minced, and incubated with 10 ml trypsin (2.5 mg/ml) and DNase (0.015 mg/ml) in serum free medium (30 min, 37°C). Tissue was triturated, resuspended in 10 ml culture medium with 10% fetal bovine serum, filtered through 135 μm and then 45-μm pore nylon mesh, prior to plating (~2.5 × 10^5 ^cells/cm^2^) on poly-D-lysine coated coverslips. After mixed glia from wild-type or *Cx3cr1*^-/- ^mice were cultured approximately 6-8 days *in vitro *they were used as a bedlayer for co-culturing neurons. Despite functional deletion of *Cx3cr1*, the null gene was engineered to continue to express GFP as a marker [see [26]]. The proportion of Hoechst^+ ^cells in the mixed glial cultures that were GFP^+ ^was determined following 48 h of continuous vehicle, morphine, HIV-1 Tat, or morphine plus Tat exposure (Table 1). At least 300-500 cells were counted per treatment group in each experiment (N = 1). Experiments were repeated 3 times and data presented as the mean ± the SEM (N = 3).

### Neuronal culture or co-culture with mixed glia from wild-type or *Cx3cr1*^-/- ^(*Cx3cr1*^GFP/GFP^) mice

Striatal neurons were prepared from wild-type E15-E16 ICR (CD-1) mouse embryos. Striata were dissected, minced, and incubated with 10 ml trypsin (2.5 mg/ml) and DNase (0.015 mg/ml) in Neurobasal medium (Invitrogen, Carlsbad, CA) with 25 μM glutamate (30 min, 37°C). Tissue was triturated, resuspended in 10 ml medium, and cells filtered twice through 70 μm pore nylon mesh. Isolated wild-type neurons were (i) plated alone on poly-D-lysine coated coverslips or (ii) co-cultured directly on a bedlayer of mixed glia derived from wild type or *Cx3cr1 *^-/- ^mice. Neuron cultures and neuron-glia co-cultures were similarly maintained in Neurobasal medium supplemented with B27, 0.5 mM L-glutamine, 0.025 mM glutamate, and allowed to mature for about 1 wk prior to the start of the experiments.

### Assessment of neuron viability

Time-lapse digital images were recorded at 20 min intervals for 48 h using a microscope (Zeiss Axio Observer Z.1) and automated, computer-controlled (AxioVision 4.6; Mark&Find, MosaiX, and Time Lapse software) stage encoder with environmental control (37°C, 95% humidity, 5% CO_2_; PeCon Instruments, Houston, TX), to repeatedly track the same neurons [64]. Repeated measures ANOVA was used to compare treatment effects on the same neuron at the onset and throughout the experiment (Statistica; StatSoft, Tulsa, OK). In each experiment, treatments were distributed across cells pooled from the same pups. Approximately 50 healthy neurons with well defined dendritic and axonal arbors were identified using phase-contrast microscopy (40× magnification) within individual culture wells in each experiment prior to treatment (0 h) (Figure 2A-C). The effects of opioids and/or HIV-1 Tat ± fractalkine or CX_3_CR1 immunoblockade ± mixed glia from wild-type or *Cx_3_cr1*^-/- ^mice on neuron survival was determined at 4 h intervals and averaged per culture well/treatment (n = 1). Neuron injury and death was monitored in individual cells and defined by rigorous criteria, which included dendritic pruning, dissolution of the Nissl substance, transient cytoplasmic swelling and vacuolization, nuclear damage, and eventual destruction of the cell body [61,63,64]. In some cases, neuron death was confirmed by viability markers such as ethidium homodimer, ethidium monoazide, or trypan blue (not shown); however, these markers all possess some inherent cytotoxicity with prolonged exposure, which precludes their use to monitor cells continuously. The effect of each treatment on neuron survival was analyzed statistically at 4 h intervals using repeated measures ANOVA from n = 5-6 experiments (200-300 neurons in total per treatment) and reported as the mean percentage of surviving neurons, relative to pretreatment values ± the standard error of the mean (SEM), but designated as "% of control" for simplicity.

### Experimental treatments

Cultures were continuously treated with recombinant Tat_1-86 _(100 nM; from HIV-1_IIIB _strain, ImmunoDiagnostics, Woburn, MA), morphine sulfate (500 nM; Sigma, St. Louis, MO), full-length recombinant mouse fractalkine (1 μg/ml) (R&D Systems, Inc., Minneapolis, MN; catalog no. 472-FF/CF), and/or anti-CX_3_CR1 antibodies lacking azide (Torrey Pines Biolabs, East Orange, NJ).

### Immunocytochemistry

Cell cultures were fixed for 15 min in 3% paraformaldehyde in phosphate buffer (pH 7.2 at 4°C), permeabilized in 0.1% Triton-X 100, and rinsed 3 × 20 min in PBS, pH 7.2. Cultures were incubated in diluted primary antiserum on an orbital shaker (40-60 rpm) overnight at 4°C in PBS, pH 7.2 with 1% crystalline grade BSA (Calbiochem/EMD Chemicals, Gibbstown, NJ) and 0.1% Triton-X 100. Rabbit anti-mouse fractalkine (1:100; catalog no. TP233; Torrey Pines Biolabs) or rabbit anti-rat CX_3_CR1 (1:100; catalog TP501; Torrey Pines Biolabs) antigenicity was co-localized with antibodies to MAP2 a/b (1:200; catalog MAB 378; Chemicon, Temecula, CA), glial fibrillary acidic protein (GFAP) to detect astroglia (1:200; Chemicon), or CD11b (1:100; catalog No. MAB1124; R&D Systems) to identify microglia. MAP2, GFAP, and CD11b antigenicity were detected using appropriate secondary antibodies conjugated to Alexa 488 (1:500 dilution; Molecular Probes, Eugene, OR) or Cy3 (1:250 dilution; Jackson ImmunoResearch, West Grove, PA). Cells were counterstained with Hoechst 33342 (1 μg/ml), which labels all cell nuclei blue. A rabbit polyclonal antibody against Iba-1 (Wako Chemicals USA, Richmond, VA, 1:500) and an anti-rabbit secondary antibody conjugated to Alexa 594 were also used to detect microglia and ascertain the extent of Iba-1-GFP overlap in mixed glial cultures from *Cx3cr1*^-/- ^mice. Cultures were rinsed and mounted in Prolong Antifade compound (Molecular Probes). For measurements of dendrite length, neuron cell bodies and dendrites were labeled using anti-MAP2a/b antibodies (1:200; Chemicon) and secondary antibodies conjugated to horseradish peroxidase and visualized using diaminobenzidine. Preabsorbed fractalkine antibody controls consisted of rabbit anti-murine fractalkine antibody (R&D Systems) IgG preabsorbed with 25 μg/ml of the recombinant fractalkine peptide epitope used for antibody production for 1 h at 37°C, 5% CO_2 _and in high humidity.

### Fluorescence microscopy

Fluorescent images were obtained using Zeiss Axio Observer Z.1 microscopes configured with widefield fluorescence and AxioCam MRm digital camera or configured with a LSM 700 laser scanning confocal head. Multiple z-stacks were acquired and compressed into a single projected image to better show the cells in their entirety using Zen 2010 software (Zeiss).

### Immunoblotting

Western blotting for fractalkine and CX_3_CR1 was performed using routine methods previously reported by us [62,64]. Goat anti-mouse fractalkine antibodies (directed against the N-terminal, chemokine domain; catalog no. AF472; R&D Systems) were used at 1:1,500 dilution and rabbit anti-rat CX_3_CR1 antibodies (TP501; Torrey Pines Biolabs) were used at 1:1,000 dilution. The same non-glycosylated, recombinant fractalkine that was neuroprotective against morphine and/or Tat co-exposure (R&D Systems; catalog no. 472-FF/CF) served as a positive control. Proteins were detected via HRP and enhanced chemoluminescence detection (Amersham Biosciences, Fairfield, CT), and quantified using a Kodak 440 Gel Imager. Protein levels were normalized to β-actin (1:3,000; Santa Cruz Biotechnology, Santa Cruz, CA).

### ELISA

Fractalkine and TNF-α were assessed by ELISA (R&D Systems) according to the manufacturer's instructions using a Victor-3 platereader (Perkin Elmer Co., Waltham, MA) as previously described [64,70].

### Microglial migration

Individual microglia were tracked and images acquired at 20 min intervals, analyzed at 1 h intervals, and the average movement determined during the initial 12 h following experimental treatments. Microglia are readily identified in videos as motile, amoeboid cells with cytoplasmic granularity caused by lysosomes and autophagosomes, and filopodial/lamellipodial cytoplasmic extensions. Microglial identification was verified by immunostaining for Iba-1 at the end of some experiments (Figure 4B). Microglia were randomly selected from within the phase-contrast images at 0 h and the point-to-point distance traveled by each macrophage/microglia was assessed at 1 h intervals, by measuring the linear distance from the last position of the cell body. Because most cells tended not to move in a straight line at a consistent speed, even measurements at relatively frequent 1 h intervals underestimated the total distance traveled by many cells during that time. Despite some irregular movement by individual microglia, the average distance traveled per unit time by all microglia within a particular treatment group was quite reproducible. Approximately 100 microglia were selected in each treatment group per experiment. Cells that moved out of the viewing field were excluded from the determination. Data are presented as the mean migration rate (μm/h) ± SEM from 4 experiments.

### Dendrite length estimates

After the time lapse studies were completed at 72 h, cultures were fixed for 15 min with ice-cold Zamboni's fixative, washed in PBS (3 × 5 min), and incubated with anti-rabbit microtubule associated protein-2 (MAP2) antibodies overnight at 4°C (1:200 dilution; Chemicon). MAP2 immunofluorescence was detected using biotinylated secondary antibodies conjugated to peroxidase (Jackson ImmunoResearch, West Grove, PA) and visualized using diaminobenzidine as a peroxidase substrate. The length of all dendrites within a defined area was estimated by their intersections with calibrated concentric circles using the Scholl procedure [94,95], but with slight modifications for *in vitro *measurements [61]. The mean dendritic length per neuron was calculated from approximately 40-60 neurons sampled per treatment group in each experiment; experiments were repeated at least 3 times and data presented as the mean dendritic length per neuron ± SEM per experiment.

### Statistical analyses

Statistical analyses were done by repeated measures, one-way, and two-way analysis of variance (ANOVA) followed by Duncan's or Bonferroni's post-hoc testing (StatSoft, Statistica, Tulsa, OK).

## List of Abbreviations

ADAM: a disintegrin and metalloproteinase; ANOVA: Analysis of variance; RGD domain: Arg-Gly-Asp domain; CCL2 (MCP-1): chemokine (C-C motif) ligand 2 or monocyte chemoattractant protein-2; CCL5 (RANTES): chemokine (C-C motif) ligand 5; ELISA: enzyme-linked immunosorbent assay; ERK: extracellular signal-regulated kinases; [Ca^+2^]_o_: extracellular calcium; CX_3_CR1: fractalkine receptor; CX_3_CL1: fractalkine; GFAP: glial fibrillary acidic protein; iNOS: inducible nitric oxide synthase; IL-1β: interleukin-1β; [Ca^+2^]_i_: intracellular calcium; Iba-1: ionized calcium binding adaptor molecule 1; HAND: HIV-associated neurocognitive disorders; HIV-1: human immunodeficiency virus-type 1; MAP-2: microtubule-associated protein type-2; MCP-1: monocyte chemoattractant protein-1; MAPK: mitogen-activated protein kinase; NK cells: natural killer cells; neuroAIDS: neuro-acquired immunodeficiency syndrome; NADPH: nicotinamide adenine dinucleotide phosphate oxidase; PI3K: phosphoinositide 3-kinase; RANTES (CCL5): regulated upon activation, normal T-cell expressed, and secreted; Tat: transactivator of transcription; TNF-α: tumor necrosis factor-α; TACE (or ADAM 17): TNF-α converting enzyme.

## Competing interests

The authors declare that they have no competing interests.

## Authors' contributions

MS, JLS, DHC, PEK, and KFH designed the experiments. MS, SZ, NE-H, Y-KH, and MES conducted the experiments. MS, JLS, DHC, PEK, and KFH analyzed and interpreted the data and wrote the manuscript. KFH coordinated the research and supervised the project. All authors have read and approved the final manuscript.
